# Role of regenerating gene IA expression on local invasion and survival in nasopharyngeal carcinoma

**DOI:** 10.1186/s40659-017-0142-7

**Published:** 2017-11-21

**Authors:** Haijie Xing, Xiangdong Chen, Yaofeng Han

**Affiliations:** 1 Department of Otorhinolaryngology Head and Neck Surgery, Guang Ming New District People’s Hospital, No.4253 Songbai rode, ShenZhen, 518106 China; 2 Department of Otorhinolaryngology Head and Neck Surgery, The Affiliated Xinhua Hospital, Hainan Medical College, Haikou, 570311 China; 3 Department of Otolaryngology Head and Neck Surgery, Affiliated Hospital of Shenzhen University, Shenzhen, 518055 Guangdong Province China; 4 Department of Epidemiology, Public Health College of Xiamen University, Xiamen, 361005 Fujian China

**Keywords:** Nasopharyngeal carcinoma, Regenerating gene IA, Invasion, Survival

## Abstract

**Background:**

Regenerating gene IA (REGIA) plays an important role in tissue regeneration and tumors prognosis of epithelium origin. However, the role of REGIA in nasopharyngeal carcinoma (NPC) is unclear. This study aims to investigate the expression and function of REG1A in NPC.

**Results:**

We have found that there was 63 patients with REGIA positive expression of 155 patients in this study (40.65%). The positive expression rate of REGIA was 30.50, 44.44 and 47.83% in stage T2, T3 and T4 patients, respectively. The REGIA expression was significantly difference in T2 and T4 stage tumors or T2 and T3–T4 stage. The positive expression rate of REGIA was found to be higher in patients with cervical lymph node persistence than those with cervical lymph node complete regression. Patients with negative REGIA expression had a better overall survival and free survival than those with REGIA positive expression. In addition, according to the univariate and multivariate analysis, the REGIA expression was an independent adverse prognostic factor for NPC patients.

**Conclusion:**

REGIA expression was a useful biomarker in NPC patients for assessing T stage and survival.

## Background

Nasopharyngeal carcinoma (NPC) is an endemic disease in southern parts of China [1]. Approximately 70% of newly NPC patients present with stage III or IV disease [2]. Radiotherapy with or without concurrent chemotherapy is a standard modality [3]. Although many advancements has been made over the past decades in radiotherapy technology, the reason for treatment failures included disease persistence, residues, local-region recurrence and distant metastases which were unfavorable prognostic factors [4]. Studies indicated that NPC progression and prognosis was a genetically controlled [5, 6]. Thus, to find a biomarker in NPC patients was necessary which could assess the treatment and prognosis in clinical practice.

Regenerating gene (Reg) was originally isolated from a complementary DNA (cDNA) library derived from regenerating rat pancreatic islets, and its human homologue was named as REGIA [7]. Until now, 17 members of the Reg family have been identified and classified into four classes (Reg I–IV) [8], consisting of acute phase reactants, lectins, anti-apoptotic factors or growth factors for pancreatic islet cells and epithelial cells in the digestive system [9].

Regenerating gene IA (REGIA) is a sub-classification of human REG I gene, encoding a 166-amino-acid protein with a 22-amino-acid signal sequence, which is predominantly expressed in human pancreatic secretion [10]. In human, the REGIA, along with other members of REG I gene, such as REG1B, REG-related sequence and PAP, are clustered tandemly in a 95-kb region of chromosome 2p12. Recently, REGIA was found to be involved in not only the inflammatory diseases [11] but also the various gastroenterological cancers [12]. REGIA also plays an important role in various tumor progression and recurrence, such as breast cancer [13], lung cancer [14], bladder cancer [15], colorectal cancer [16] and so on. Furthermore, several studies reported that REGIA expression was associated with cross-reaction in esophageal squamous cell cancer treatment [17].

The present study aim to investigate the role of REGIA expression in NPC, where we retrospectively detected REGIA expression as well as its association with clinic-pathological factors and prognosis of NPC. The result showed that the NPC tissues expressing REGIA was associated with NPC local invasion and poor survival, which suggested that REGIA could serve as a biomarker of NPC patients in diagnosis and detection of unfavorable prognosis.

## Methods

### Patients

The information of 183 patients from February 2010 and October 2015 in the Affiliated Xinhua Hospital of Hainan Medical College were collected. There was 155 cases with complete information that included follow up records and sufficient specimen samples of NPC underwent definitive radiotherapy with or without chemotherapy were chosen for the research. The 155 patients contained 147 males and 8 females, and their mean age was 60.3 ± 0.73 years (range 34–81 years). Basing on the recent World Health Organization (WHO) classification systems [18], the pathologic type in this study was distributed as follows: the WHO type II included two pathologic type, the differentiated that contained 4 patients (2.6%) and undifferentiated that contained 151 patients (97.4%). According to the Tumor-Node-Metastasis (TNM) classification [19], there was 59 cases (38%) diagnosed with T2 NPC, 27 cases (17.4%) with T3 NPC, and 69 cases (44.5%) with T4 NPC respectively. There was 30 cases (19.4%) diagnosed as N1 disease, 32 cases (20.6%) diagnosed as N2, 8 cases (5.2%) diagnosed as N3, respectively. Forty-eight patients were diagnosed with Stage II, 36 patients with stage III and 71 patients with stage IV, respectively (Table 1).

**Table 1:** Characteristics of the patients in the study

Characteristics	All patients (n = 155)	REG IA expression		P value
		Positive (n = 63)	Negative (n = 92)	
Age (years)				0.348
≤ 60	71	26	45	
≥ 61	84	37	47	
Gender				1.000
Female	147	60	87	
Male	8	3	5	
WHO histologic type				0.246
Differentiated	4	0	4	
Undifferentiated	151	63	88	
T stage				0.049
T2	59	18	41	
T3	27	12	15	
T4	69	33	36	
N stage				0.589
N0	85	33	52	
N1	30	13	17	
N2	32	12	20	
N3	8	5	3	
Clinical stage				0.067
II	48	13	35	
III	36	16	20	
IV	71	34	37	

### Treatment modality

All patients undergone radical radiotherapy at their primary site, with the mean radiation dose of 72.89 Gray (range 70–78 Gray) in two fractions daily using lateral parallel pair radiation for 7–8 weeks. Ninety-six patients were treated with conventional radiotherapy while 59 with an accelerated schedule. The lymph nodes were delivered with a mean of 67.20 Gray (range 60–78 Gray). There was no significant difference between the patients received conventional radiotherapy and accelerated schedule.

Seventy-six patients were subjected to chemotherapy, of which 17 patients received neoadjuvant chemotherapy that mainly consisted of 2–3 cycles of PF (cisplatin 30 mg/m^2^/day IV for 3 days, 5-FU 800–1000 mg/m^2^ IV in d1–d5) at an interval of 2 weeks prior to the initiation of radiotherapy treatment. And there was 42 patients received 2–3 cycles of PF (cisplatin 30 mg/m^2^/day IV for 3 days, 5-FU 800–1000 mg/m^2^ IV in d1–d5) at an interval of 2 weeks given concomitantly with radiotherapy. Remaining 17 patients received 3 cycles of chemotherapy (cisplatin 100 mg/m^2^) given concomitantly with radiotherapy at days 1,4,7,10,13, and 16. After finishing radio-chemotherapy, 11 patients (3 of neoadjuvant chemotherapy, 8 of concurrent chemo-radiotherapy) received a chemotherapeutic regimens that consisted of cisplatin (80 mg/m^2^) at day 1 and 5-fluorouracil (1000 mg/m^2^ per day) during days 1–5 for every 4 weeks for 2–3 cycles.

### Immunohistochemistry and staining assessment

155 paraffin-embedded NPC pre-treated biopsy sample blocks were collected from the histology laboratories at the Affiliated Xinhua Hospital. The slides having a thickness of 4 μm were sliced from the blocks. The tissue sections were deparaffinized in xylene, following by rehydration in a graded series of ethanol solution, and finally washed with phosphate-buffered saline (pH7.4).

(1) Antigen retrieval was carried out by keeping in microwave with 10 mmol/l citrate buffer (pH6.0) for 25 min followed by blocking of the endogenous peroxidase by immersing the slides in 250 ml methanol containing 2.5 ml hydrogen peroxide solution for 30 min. (2) Anti-REGIA mouse monoclonal antibody (Q01, Lifespan, USA) of 1:200 dilution in Tris-buffered saline (TBS) was added to each slide and were incubated for 1 h at room temperature followed by 5 min washes with TBS for three times. (3) Biotinylated anti-mouse secondary antibody [SP-900(general type), Zhongshan, China] at 1: 200 dilution in TBS was added and incubated for 30 min at room temperature, followed by 5 min washes with TBS for three times. (4) Finally, the sections were incubated with streptavidin–biotin complex at 1:500 for 30 min at room temperature. Then it was washed with TBS, and finally stained with 3, 3-diaminobenzidine. (5) After counterstaining with hematoxylin, the slides were dehydrated and mounted for visualisation. (6) Substitution of the primary antibody with the identical concentration of mouse IgG1 (ZA-0448, Zhongshan, China) served as a negative control. Batch-to-batch variation was assessed by choosing two sections showing high and low REGIA expression and running additional sections from these biopsy samples with each batch.

Immunostaining results were evaluated and scored blindly and independently by two pathologists, with resolution of any conflicting scores being done by discussion and consensus. REGIA staining results were scored into four levels according to the percentage of cytoplasmic positive tumor cells in 10 high power fields for each slide: 0: less than 5%, 1: 6–25%, 2: 26–50%, 3: more than 50%. Similarly, staining intensity was assigned a score as follows: 0 = no staining, 1 = weak staining, 2 = moderate staining, 3 = strong staining. The two individual parameters were added, resulting in an immunoreactivity score (IRS) ranging from 0 to 6. We defined cases with IRS > 4 as positive expression, and cases with IRS ≤ 4 as negative expression [20]. This study was approved by the Research Ethics Committee of the Affiliated Xinhua Hospital, Hainan Medical College. Written informed consent was obtained from every patient.

### Statistical analysis of data

The primary endpoints of this study were treatment completion, the secondary endpoints were to detect disease progression (newly occurred metastatic lesion, persistence, recurrence or expansion of the primary and/or region lesion), death or last follow-up. The Overall Survival (OS) was calculated from the first day of the completion of treatment till death or the end of follow-up, and Progression Free Survival (PFS) was calculated from the completion of treatment to the date of disease progression.

The patients were divided into REGIA positive or negative using the Chi square tests. Cumulative incidence method was used to estimate the relationship of REGIA expression in tumor cells with overall survival and progression-free survival. The cumulative incidence curves were compared by the log-rank test using Kaplan–Meier method. Univariate and multivariate analysis were performed with a Cox proportional hazards model for overall survival and progression-free survival to determine the effect of REGIA expression in univariate analysis that retained significance after covariates adjustment. P < 0.05 (two-sided tests) was considered to be significantly. Relative risks were presented with 95% confidence intervals. SPSS statistical package (Version 19.0, SPSS Inc., Chicago, IL) was used for all the analyses.

## Results

### REGIA expression and its relation to patient’s characteristics distribution

**Fig. 1:**
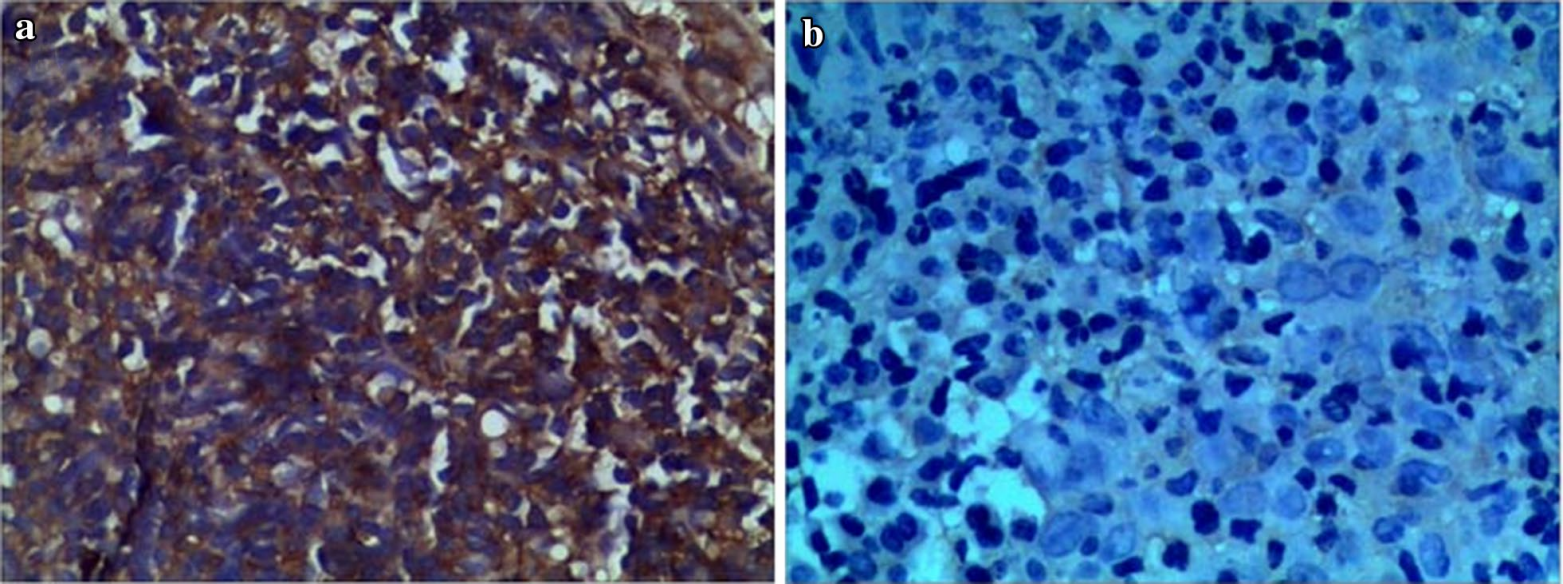
REG IA expression in tumor cell cytoplasm. **a** Representative immuno-staining of REG IA expression positive. SP. 400. The REG IA expression in the cytoplasm. **b** Representative immuno-staining of REG IA expression negative. SP. 400

The 155 patients were analyzed basing on immuno-histochemical, and the results showed that 63 cases (40.65%) were REGIA positive expression (Fig. 1a), and 92 cases were REGIA negative expression (Fig. 1b).

The positive rate of REGIA expression presenting in patients at stage T2, stage T3 and stage T4 were 30.50, 44.44 and 47.83%, respectively. And there was a significant difference in the REGIA expression between T2 stage and T4 stage tumors, and T2 stage and T3–T4 stage tumors. These results indicated that patients with REGIA positive expression have higher T stage than those with REGIA negative expression. In addition, there was no significant correlation between REGIA expression and age, gender, WHO classification, N stage and clinical stage (Table 1).

### Relationship between REGIA expression and survival

The tumor progression was detected in 37 out of 155 (23.87%) patients during follow-up (the median follow-up period was 33.9 months, range from 3.3 to 131.9 months). Of which, 7 patients relapsed in primary site, and 15 patients relapsed in cervical region. Among them 3 cases and 10 cases were reported to be REGIA positive expression. One and twenty-four patient were progressive in primary site and in cervical lymph node, respectively, and nineteen patients were progressive in both primary site and cervical lymph node. Moreover, there were 14 of 24 cases and 17 of 19 cases who were reported to REGIA positive expression. The data showed that positive expression rate of REGIA in patients with cervical lymph node disease progression are higher than those with cervical lymph node disease complete regression, P < 0.05. These results suggested that patients with REGIA positive expression are more subjected to cervical lymph node disease progression.

**Fig. 2:**
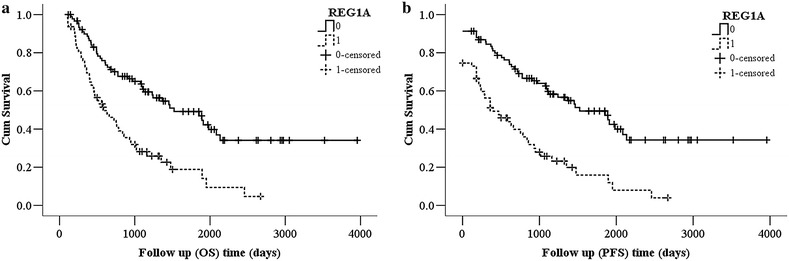
The effect of REG IA expression on the survival. **a** Cumulative incidence (CI) curves showing the relationship between tumor REG IA expression and OS. **b** Cumulative incidence (CI) curves showing the relationship between tumor REG IA expression and PFS. Straight line REG IA expression negative, dashed line REG IA expression positive

As shown in Fig. 2a, b, among REGIA negative and positive expression cases, the OS rate in 5 years were 49.18 and 18.84% respectively, Log Rank = 20.78, P = 0.0000, and the PFS rate were 49.44 and 15.90% Log Rank = 25.41, P = 0.0000, suggesting that the patients with REG1A positive expression have a poor survival.

### Relationship between REGIA expression and OS or PFS

Considering age, gender, WHO classification, T stage, N stage, clinical stage, and REGIA expression, the Univariate and multivariate analyses showed that REGIA expression is a dependent prognostic factor for NPC patients, implying that REGIA expression could be a useful prognostic factor in NPC patients (Tables 2 and 3).

**Table 2:** Univariate and multivariate analyzing outcomes for OS

Parameters	Univariate		P value	Multivariate		P value
	RR	95% CI		RR	95% CI	
Age	1.206	0.802–1.815	0.369	0.860	1.533–3.580	0.485
Gender	0.904	0.285–2.865	0.863	0.963	0.298–3.111	0.949
Tumor differentiation	0.047	0.000–9.757	0.262	0.000	0.000–1.33 + 228	0.967
T stage	1.681	1.326–2.132	0.000	1.555	0.830–2.914	0.168
N stage	1.452	1.193–1.767	0.000	1.264	1.005–1.590	0.046
Clinical stage	1.804	1.398–2.329	0.000	1.021	0.497–2.096	0.956
REG1A expression	2.525	1.673–3.810	0.000	2.343	1.533–3.580	0.000

*RR* Relative risks, *CI* Confidence interval, *REGIA* Regenerating gene IA

**Table 3:** Univariate and multivariate analyzing outcomes for PFS

Parameters	Univariate		P value	Multivariate		P value
	RR	95% CI		RR	95% CI	
Age	1.130	0.708–1.803	0.609	0.866	0.537–1.396	0.554
Gender	0.399	0.055–2.885	0.363	0.515	0.070–3.765	0.513
Tumor differentiation	0.047	0.000–15.615	0.302	0.000	0.000–3.87 + 239	0.968
T stage	1.555	1.193–2.026	0.001	1.428	0.682–2.991	0.344
N stage	1.254	0.982–1.602	0.069	1.062	0.797–1.415	0.683
Clinical stage	1.642	1.241–2.173	0.001	1.114	0.484–2.564	0.799
REG1A expression	2.652	1.649–4.266	0.000	2.617	1.596–4.293	0.000

*RR* Relative risks, *CI* Confidence interval, *REGIA* Regenerating gene IA

## Discussion

Our studies have shown that REGIA was expressed predominantly in NPC tissue, and the tissue and structures that adjacent to these tumors were prone to invasion and had a poor survival. Tumor invasion scores were verified by the radiation oncologists who appreciated the treatment modality and conferred accurate field of radiation to ameliorate prognosis. Astrosini et al. [16] reported that REGIA expression level was significantly increased in colorectal cancer with peritoneal carcinomatosis. Another study also suggested that hepatocellular carcinoma with elevating REGIA expression had shown more frequent high-stage tumors than the hepatocellular carcinoma showing PAP expression alone [21].

In current study, 63 of 155 patients were found to be REGIA positive expression. The increase in REGIA positive expression rate (30.50, 44.44 and 47.83%) along with the T stage of NPC were becoming high in T2, T3 and T4. The REGIA positive expression rate in T2 stage was found to be lower than those in stage T3–T4 or T4. Patients with cervical lymph node disease progression also have a higher REGIA positive expression rate than those with cervical lymph node disease complete regression after treatment. These results indicate that REGIA expression plays an important role in the invasion and metastasis of NPC.

Certain studies had explored the underlying molecular mechanism of REGIA expression in the tumor’s infiltration and metastasis. In pancreatic cancer cell lines and colitic cancer, overexpression of REGIA resulted in accelerated cell proliferation and consequently tumor growth [22]. Sekikawa et al. [23] confirmed REGIA was an important downstream molecule of STAT3 signaling pathway which mediated the anti-apoptotic effect by activating the Akt/Bad/Bcl-xL pathway. The addition of anti-REGIA antibody clearly inhibited anti-apoptotic action of REGIA. Cavard et al. [24] reported that REGIA was a downstream target of the Wnt/b-catenin pathway. REGIA could in turn promote Akt phosphorylation and upregulate the anti-apoptotic gene Bcl-xL and Bcl-2 expression [25, 26]. In addition, cytokines, interferon gamma, interleukin 6, and interleukin 22, were reported to be contribute to REGIA transcription [27, 28]. As a consequence, increased REGIA expression could promote tumors cell proliferation and reduce apoptosis through multiple pathways. REGIA expression was found to be associated with the poor prognosis in a variety of tumors [29]. The 10-year disease-specific survival rate among patients with lower levels of REGIA was significantly better than those with higher levels, and the REGIA could independently affect the survival rate in patients with breast cancer [13]. The present study found that REGIA positive expression was associated with poor survival in NPC. Patients with REGIA negative expression had a higher OS and PFS rate than those with REGIA positive expression.

Researchers speculated that the poor prognosis was due to the induction of tumor cell proliferation, differentiation and tissue regeneration by REGIA. The possible potential mechanisms for REGIA activity included: First, REGIA expression may inhibit the tumor cell apoptosis through acceleration of STAT3 and Bcl-2 expression [30]. Secondly, REGIA, being a downstream factor, may participate in the Wnt signaling pathway and expedite cell proliferation and differentiation [24]. Finally, REGIA regulated the scale factor of bcl-2/bcl-xl [31].

Both univariate and multivariate analysis demonstrated that REGIA expression was associated with OS and PFS, and conducted harmful effects on NPC progression and survival. This study suggested that REGIA expression could serve as an independent adverse factor for survival in NPC. Thus, more attention should be paid in identifying the role of REGIA in the NPC, which was intended to explore molecular mechanism of invasion and metastasis in NPC, and early diagnosis to improve patient’s outcome in future. REGIA expression may serve as a reliable marker for predicting T stage and survival in NPC.

## Abbreviations

NPC: nasopharyngeal carcinoma
REGIA: regenerating gene IA
Reg: regenerating gene
